# Opening research sites in multicentre clinical trials within the UK: a detailed analysis of delays

**DOI:** 10.1186/1745-6215-14-S1-O59

**Published:** 2013-11-29

**Authors:** Paula Williamson, Carrol Gamble, Anna Kearney, Helen Hickey

**Affiliations:** 1 University of Liverpool, Liverpool, UK

## Background

The challenges of quickly obtaining the necessary research approvals to open multisite clinical trials in the UK, is well documented.

This presentation takes a fresh, in depth look at the factors currently limiting the efficient set up of multicentre clinical trials. We seek to discover whether newly imposed timeframes and penalties aimed at improving the speed of local NHS R&D governance checks have had the desired impact, and if so, what other activities are impacting the time taken to open research sites.

## Methods

We present a case study of two trials, SANADII, a CTIMP trial aiming to recruit 1500 participants across 100+ sites and BASICS, a medical device trial, aiming to recruit 1200 participants over 17 sites. Both trials seek to have research sites across all four UK constituent nations.

Primary, secondary and tertiary delays affecting progress were recorded for sites from ethical approval to opening to recruitment. Key milestones such as SSI submission, gaining R&D approval, signing contracts and initiation training were also recorded for each site along with overarching trial milestones.

We identified sites that encountered significant delays for further analysis, looking at which milestones and activities regularly contributed to delays.

## Results

Early data suggests local research governance checks may no longer be such a limiting factor, but that other administration, document collection, and accessibility of research staff contribute significant delays.

Full analysis of the results will be presented with suggestions of how clinical trial governance should be developed to minimise impact of the reoccurring delays identified.

